# *SOX2* dosage sustains tumor-promoting inflammation to drive disease aggressiveness by modulating the *FOSL2/IL6* axis

**DOI:** 10.1186/s12943-023-01734-w

**Published:** 2023-03-17

**Authors:** Abdel Jelil Njouendou, Tibor Szarvas, Arnol Auvaker Zebaze Tiofack, Rovaldo Nguims Kenfack, Pamela Derliche Tonouo, Sidonie Noa Ananga, Esther H. M. Dina Bell, Gustave Simo, Jörg D. Hoheisel, Jens T. Siveke, Smiths S. Lueong

**Affiliations:** 1 Department of Biomedical Sciences, Faculty of Health Sciences, University of Buea, Buea, South West Region Cameroon; 2 Phytopharmacy and Drug Discovery Section, The Cameroon Consortium for Translational Cancer Research (CCOTCARE), Douala, Cameroon; 3 Department of Urology, West German Cancer Center, University Hospital Essen, University Duisburg-Essen, 45122 Essen, Germany; 4 Department of Urology, Semmelweis University, 1082 Budapest, Hungary; 5 Molecular Parasitology and Entomology Unit (MPEU), Department of Biochemistry, Faculty of Science, University of Dschang, Dschang, West Region Cameroon; 6 Early Detection and Biomarker Section, The Cameroon Consortium for Translational Cancer Research (CCOTCARE), Douala, Cameroon; 7 Research Unit of Microbiology and Antimicrobial Substances (RUMAS), Department of Biochemistry, Faculty of Science, University of Dschang, Dschang, West Region Cameroon; 8 Faculty of Medicine and Pharmaceutical Sciences, University of Douala, Douala, Littoral Region Cameroon; 9 Department of Medical Oncology, Douala General Hospital, Douala, Littoral Region Cameroon; 10 Epidemiology and Clinical Trial Section, The Cameroon Consortium for Translational Cancer Research (CCOTCARE), Douala, Cameroon; 11 Directorate of Scientific Affairs, The Cameroon Consortium for Translational Cancer Research (CCOTCARE), Douala, Cameroon; 12 Division of Functional Genome Analysis, German Cancer Research Center (DKFZ), Im Neuenheimer Feld 580, 69120 Heidelberg, Germany; 13 Bridge Institute for Experimental Cancer Therapy, West German Cancer Center, University Hospital Essen, Hufeland Str. 55, 45147 Essen, Germany; 14 Division for Solid Tumor Translational Oncology German Cancer Research Center (DKFZ)West German Cancer Center, The German Consortium for Translational Cancer Research (DKTK), Essen/Düsseldorf Partner, Site University Hospital Essen, Essen, Germany

**Keywords:** Tumor aggressiveness, Somatic copy number alterations, *SOX2*, *IL6*, *FOSL2*, Inflammation, Gene expression, Mutational signatures

## Abstract

**Background:**

Inflammation is undoubtedly a hallmark of cancer development. Its maintenance within tumors and the consequences on disease aggressiveness are insufficiently understood.

**Methods:**

Data of 27 tumor entities (about 5000 samples) were downloaded from the TCGA and GEO databases. Multi-omic analyses were performed on these and in-house data to investigate molecular determinants of tumor aggressiveness. Using molecular loss-of-function data, the mechanistic underpinnings of inflammation-induced tumor aggressiveness were addressed. Patient specimens and *in vivo* disease models were subsequently used to validate findings.

**Results:**

There was significant association between somatic copy number alterations (sCNAs) and tumor aggressiveness. *SOX2* amplification was the most important feature among novel and known aggressiveness-associated alterations. Mechanistically, *SOX2* regulates a group of genes, in particular the *AP1* transcription factor *FOSL2*, to sustain pro-inflammatory signaling pathways, such as *IL6-JAK-STAT3*, *TNFA* and *IL17*. *FOSL2* was found overexpressed in tumor sections of specifically aggressive cancers. In consequence, prolonged inflammation induces immunosuppression and activates cytidine deamination and thus DNA damage as evidenced by related mutational signatures in aggressive tumors. The DNA damage affects tumor suppressor genes such as *TP53,* which is the most mutated gene in aggressive tumors compared to less aggressive ones (38% vs 14%), thereby releasing cell cycle control. These results were confirmed by analyzing tissues from various tumor types and *in vivo* studies.

**Conclusion:**

Our data demonstrate the implication of *SOX2* in promoting DNA damage and genome instability by sustaining inflammation via *FOSL2/IL6,* resulting in tumor aggressiveness.

**Supplementary Information:**

The online version contains supplementary material available at 10.1186/s12943-023-01734-w.

## Introduction

Genomic instability is undoubtedly a major hallmark of cancer development as it drives tumor heterogeneity and supports the adaptation of cancer cells to stress conditions leading to malignant behavior and therapy resistance [1]. Oncogenic driver mutations or inactivation of DNA repair genes are amongst the early events in malignant transformation and are involved in the process of tumorigenesis [2]. Other alterations, such as deletions, amplifications, fusions and translocations, are not of such dismal consequence. The extent to which genomic alterations shape the final tumor cell phenotype is context-dependent. Precision oncology seeks to tailor patient treatment schemes to specific molecular portraits [3]. Genomic instability therefore represents a considerable challenge to personalized oncology.

Following malignant transformation, tumors exhibit diverse clinical phenotypes. Aggressive tumors are generally undifferentiated and plastic [4]. Several molecular drivers have been proposed to drive tumor aggressiveness in different cancer entities, such as *FOSL1* expression in brain cancer [4] or *MYC* and *KRAS* gene dosage in pancreatic cancer [5]. Gene dosage can modulate disease phenotype or contribute to shaping it. In effect, alterations in gene dosage might alter expression and thus cellular homeostasis, leading to tumor development or tumor progression and therapy resistance.

Apart from somatic copy number alterations (sCNAs), early onset of cancer is associated with aggressive disease profiles, at least in some cancer entities such as gastric cancers [6]. Inflammatory tumor microenvironments (TMEs) also promote cancer metastasis and aggressiveness [7]. Inflammation activates cytidine deamination via pro-inflammatory transcription factors, such as NFkB, leading to DNA double-strand breaks and tumorigenic pathway activation by supporting the susceptibility to mutagenesis in several epithelial organs [8].

A comprehensive analysis of how these different molecular factors synergize to influence disease phenotype is still missing. In this study, an integrative multi-omic pan-cancer analysis of data from The Cancer Genome Atlas (TCGA) and the Gene Expression Omnibus (GEO) data repositories as well as data generated in-house was performed in order to identify major genomic determinants of cancer aggressiveness and the underlying mechanistic processes. We uncovered an association of sCNAs and disease aggressiveness and could demonstrate that *SOX2* gene dosage is tightly associated with disease aggressiveness. Mechanistically, *SOX2* regulates the API transcription factor *FOSL2*, which in turn supports inflammatory TME by promoting *TNFA, IL6-JAK-STAT3* and other pro-inflammatory signaling pathways to induce immunosuppression and stimulate genomic instability by activation of cytidine deamination. In effect, *SOX2* has previously been linked to tumor aggressiveness [9, 10]. Similarly, inflammation is known to be associated with disease aggressiveness, at least in some cancer entities such as breast cancer [11, 12]. Our work therefor provides the mechanistic basis of these observations as well as a cancer overarching implication of *SOX2/FOSL2* in driving cancer aggressiveness.

## Methods

### Data mining

Molecular and clinical data from 27 cancer entities available at TCGA were analyzed (Suppl. Tab. S1). Molecular and clinical information was downloaded using the TCGAbiolinks Bioconductor package. sCNA data was from Affymetrix SNP 6.0 microarray analyses compared to the hg19 genome assembly. Copy number segmentation data was downloaded only for primary tumors by specifying the sample type to “primary tumors” while data category and datatype were set to “copy number variation” and "nocnv_hg19.seg", respectively. When the total number of samples for a given cohort was larger than 250, only the first 250 cases were downloaded.

For gene expression data, the database was equally used to download Illumina HiSeq gene expression quantification data generated from primary tumors. For each tumor entity, the RNA-seq data from all available samples was downloaded.

For single-nucleotide variations as well as small insertions and deletions, MAF files containing somatic mutation data from the harmonized database were used. Somatic mutation data was preprocessed using the TCGA “muse” pipeline. MAFtools was applied to estimate tumor mutational burden as well as mutational signatures. Only the top-5 mutational signatures were retrieved.

Triplicate gene expression data from two prostate cell line derivatives CWR-R1 Control and SOX2-KO were downloaded from the gene expression omnibus. The data was generated by de Wet L, Williams A, Gillard M, Kregel S et al., 2022 (PMID: 35067686) and are available at GSE166184. The CWR-R1 cell line was chosen because of the availability of ChIP-seq data from the same cell line to investigate *SOX2* binding. ChIP-seq data derived from wildtype two prostate cancer cells lines (CWR-R1 and WA01) was downloaded from GEO. The ChIP-seq data was equally generated by de Wet L, Williams A, Gillard M, Kregel S et al., 2022, PMID: 35067686) and are available at the accession number GSE166183 Additional *SOX2* and *FOSL2* ChIP-seq data was downloaded from the encyclopedia of DNA elements (ENCODE) derived from the breast cancer cell line MCF7. Gene expression data from pancreatic normal and disease tissue was generated in-house and is available at the accession number E-MTAB-1791. Gene expression data from laser capture microdissected PAAD samples were obtained from the study by Maurer et al., 2019 (PMID 30658994).

### sCNA data analysis

Following data download, sCNA data was analyzed using the gaia Bioconductor package. To this end, probe meta-files for the hg19 genome assembly were downloaded from the Broad Institute and sex chromosome names were converted to numbers (X = 23, Y = 24). After duplicate removal, a marker matrix was filtered for common copy number alterations (CNVs) that are usually present in normal samples. The filtered marker matrix was then used to create a marker object. Copy number variation data from each tumor entity was downloaded as described above and also converted into a matrix. Copy number segment thresholds of -0.3 and 0.3 were used to delineate copy number deletions (sCNAdels) and copy number amplifications (sCNAmps), respectively. Sex chromosome names were also replaced with the respective numbers to match the marker object. Tumor-related somatic copy number alterations were then identified by running the gaia feature on the marker object and the processed CNA matrix on all samples in the CNA matrix. sCNA data was annotated using the GenomicRanges and biomaRt Bioconductor packages. The same packages were equally used to intersect and annotate sCNAs from different cancer entities. The circlize package was used to generate circus plots for sCNAs and somatic mutation data.

### Analysis of gene expression and ChIP-seq data

Raw gene expression data from TCGA and GEO were filtered and quantile normalized using the EDASeq Bioconductor package. The filtered data was then converted into a DGEList object for downstream analysis using the edgeR package. Differentially expressed genes were selected by defining a log_2_-fold change of ≥ 0.9 in the TCGA data and ≥ 1 for cell line-derived data. For all data sets, the false discovery threshold was set to < 0.05. Heat maps were then generated from the list of all differentially expressed genes in each case. The gene expression deconvolution tool TIMER (http://timer.cistrome.org/) was used to determine the proportions of immune cell infiltration in gene expression data.

For ChIP-seq data analysis, fastq files were assessed for their quality using the FastQc tool and trimmed with trimmomatic. The trimmed data was then mapped to the hg38 human genome assembly using bwa short read mapper. Reads were then filtered and used for peak calling with MACS. Bedgraph and narrow peak files were exported and annotated using the ChIPpeakAnno Bioconductor package. ChIP-seq peaks were displayed using the bioconductor package trackViewer.

Gene set enrichment analysis was performed with the GSEA algorithm from the Broad Institute for the hallmarks gene set, while the Bioconductor package PathfindR was used for KEGG pathway analysis. The significance threshold for GSEA and KEGG analysis was set to < 0.05. Genes associated with aggressive cancer phenotype were determined using the boruta feature selection package.

### Xenograft generation

Tumor sections were collected in RPMI cell culture medium supplemented with 2.5% fetal bovine serum, 10 mM HEPES and 1% penicillin and streptomycin, temporarily stored in freezing medium (90% FBS & 10% DMSO) at the sampling site and later used for model establishment. Tumor pieces of about 8 mm^3^ were implanted into the flanks of 5–6 week-old female nude mice. For each tumor, 5–6 animals were used and tumor growth was monitored until tumors reached about 1000 mm^3^. Mice were sacrificed and tumors removed for the preparation of fresh frozen tissue and FFPE sections. All animal experiments were approved by the University of Buea and the Cameroon Consortium for translational Cancer Research (CCOTCARE) animal review board and performed following international guidelines.

### Immunohistochemistry

FOSL2 staining was performed on 4 µm FFPE tumor sections. The Dako REAL Alkaline Phosphatase Detection System (Dako, Santa Clara, USA) was used. Slides were dried in an oven at 60 °C for 2 h and dewaxed in an automated dewaxing system (Leica ST5010 Autostainer XL; Leica Geosystems, Heerbrugg, Switzerland). Antigen retrieval was performed by heat-induced epitope retrieval with citrate buffer (10 mM sodium citrate, 0.05% Tween 20, pH = 6) for 15 min at 110 °C in a pressure cooker. The slides were then blocked with serum-free protein blocking solution (Dako) for 30 min and incubated with a 1:500 dilution of the *FOSL2* antibody at 4 °C overnight (HPA004817, Sigma-Aldrich). Slides were then washed three times for 5 min and incubated with secondary antibody (ZytoChem Plus (HRP) One-Step Polymer anti-Mouse/Rabbit, ZUC053-100) for 30 min at room temperature. Signals were revealed by incubation with brown chromogen development using Dako Liquid DAB + Substrate Chromogen System (K3468, DAKO). The sections were counterstained with hematoxylin, dehydrated, and mounted. Slides were digitalized with a Zeiss Axio Scanner Z.1 (Carl Zeiss, Oberkochen, Germany) at 10 × magnification.

### Statistical analysis

Patient samples were classified as early onset, if the patient was diagnosed before or at the age of 45 years (mean age at diagnosis for all entities minus one standard deviation). They were otherwise considered as late onset. Aggressive cancers (poor outcome) were considered to be entities with more than 40% of cases declared dead at the end of each study, while cancers with less than 20% of cases declared dead were considered less aggressive (better outcome). A cancer was considered to have substantial early onset, if more than 10% of cases were diagnosed before 45 years of age. All patients without age or gender information were excluded from the analysis. Similarly, for survival analysis, all patients with missing data for time to last follow-up or vital status were excluded. Survival analysis was performed using the survival and survminer R packages. Cut-off threshold for dichotomization of gene expression data was determined using the surv_cutpoint function of the survminer package. All analyses were performed with the R software environment or using Graphpad prism version 8.0.0 for Windows (GraphPad Software, San Diego, USA; www.graphpad.com).

## Results

### Somatic copy number alterations are associated with tumor aggressiveness

**Fig. 1:**
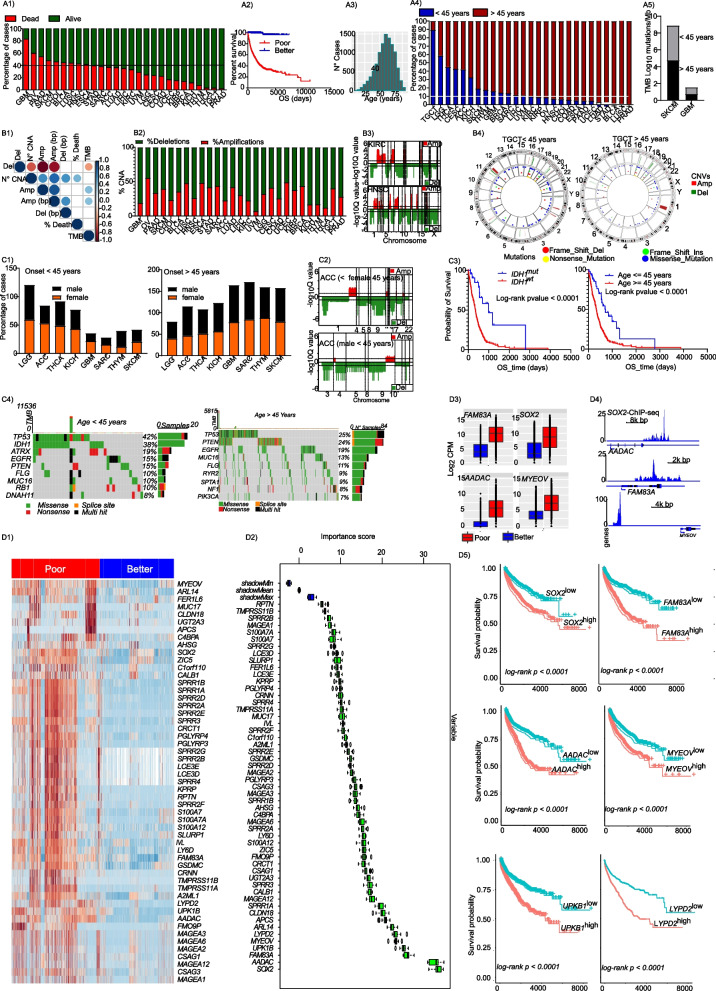
Somatic copy number alterations are associated with cancer aggressiveness. (**A1**) A bar plot presenting the percentage of cancer-related casualties in 27 cancer entities from the TCGA. Each bar represent a cancer entity and the green color represent the percentage of all patients who were reported alive at the last follow-up, while the red bars represent the percentage of patients who were confirmed dead at the last follow-up. (**A2**) A Kaplan–Meier overall survival curve for patients from the first 10 poor outcome cancer entities (red) and the last 5 better outcome entities (blue) (from Fig. 1a1). The survival time represent the time from first diagnosis to last follow-up. (**A3**) A histogram showing age distribution across all analyzed 27 cancer entities from the TCGA. The average age at first diagnosis for all cancer entities analyzed was ~ 58 years with a standard deviation of ~ 13 years. (**A4**) A bar plot presenting the fraction of patients who were diagnosed with cancer before or after 45 years. The cut-off of 45 years represents the mean age at diagnosis minus one standard deviation around the mean. (**A5**) A bar plot showing the tumor mutational burden in two of the most aggressive entities, where more than 10% of cases were diagnosed before 45 years of age. (**B1**) A correlation plot showing the correlation between the percentage of dead cases per entity and copy number alterations (CNA) as well as tumor mutational burden (TMB). The number of identified CNA (N° CNA) as well as the number of deletions (Del) or amplifications (Amp) and the number of amplified (Amp bp) or deleted base pairs (Amp bp) are presented. (**B2**) A bar plot showing the percentage of amplifications or deletion in each of the 27 cancer entities. (**B3**) representative CNV plots demonstrating higher copy number deletion. (**B4**) A circus plot showing the copy number alterations and mutations in TGCT for patients diagnosed before (left panel) and after (right panel) 45 years of age. TGCT is the entity with the highest number of patients diagnosed before 45 years of age. (**C1**) Bar plots showing the percentage of male and female cases diagnosed before the age of 45 years, for all cancer entities with more than 10% of cases diagnosed before 45 years of age. Gender-specific entities are not included here. (**C2**) Gaia CNV plots for ACC in females and males diagnosed before the age of 45 years, respectively. ACC is one of the entities with higher incidence in females before 45 years of age. (**C3**) A Kaplan–Meier overall survival curve for glioblastoma patients with and without *IDH1* mutation (left) and A Kaplan–Meier overall survival curve for glioblastoma patients diagnosed before and after the age of 45 years (right). (**C4**) An oncoprint showing the top 20 most mutated genes in glioblastoma in patients diagnosed before the age of 45 years and an oncoprint showing the top 20 most mutated genes in glioblastoma in patients diagnosed after the age of 45 years. (**D1**) A heat map showing all genes with copy number amplifications and concomitant upregulation (log fold change ≥ 0.9, FDR < 0.05) in the top 10 most aggressive tumors. (**D2**) A variable of importance box plot for all gene with copy number amplifications and concomitant upregulation in the top 10 most aggressive tumors. (**D3**) Boxplots showing the transcript expression of SOX2-regulated genes. These genes are copy number amplified in poor outcome cancer and show concomitant upregulation. (**D4**) Accumulation of SOX2 peaks around its targets genes amplified and upregulated in poor outcome cancers. (**D5**) Kaplan–Meier overall survival plots for the top most significant genes in the multivariate model

We investigated the molecular traits of tumor aggressiveness in 27 cancer entities, whose data were available at TCGA. Molecular data from highly aggressive cancers (> 40% fatalities) and less aggressive cancers (< 20% fatalities) were compared. Based on this criteria, 10 cancer entities: Glioblastoma multiforme (GBM), Pancreatic adenocarcinoma (PAAD), Ovarian serous cystadenocarcinoma (OV), Skin Cutaneous Melanoma (SKCM), Cholangiocarcinoma (CHOL), Bladder Urothelial Carcinoma (BLCA, the TCGA cohort is predominantly muscle-invasive BLCA), Lung squamous cell carcinoma (LUSC), Head and Neck squamous cell carcinoma (HNSC), Esophageal carcinoma (ESCA) and Stomach adenocarcinoma (STAD) were categorized as highly aggressive. The following tumor entities were classified as less aggressive: Prostate adenocarcinoma (PRAD), Thyroid carcinoma (THCA), Thymoma (THYM), Testicular Germ Cell Tumors (TGCT), Kidney Chromophobe (KICH), Kidney renal papillary cell carcinoma (KIRP) and Breast invasive carcinoma (BRCA) (Fig. 1, A1). More than 50% of patients with aggressive tumors had died within 2,500 days of follow-up, while more than 90% of patients with less aggressive tumors were alive after 10,000 days of follow-up (Fig. 1, A2). Mean age at diagnosis of all cancer entities was 58 ± 13 years (Fig. 1, A3). There was no direct relationship between age at diagnosis and fatality percentage; some less aggressive tumors, such as PRAD, were found exclusively in older patients (Fig. 1, A4). In some highly aggressive tumors, such as SKCM, high tumor mutational burden was observed both in young and older patients at comparable proportions. However, comparable but lower tumor mutational burden (TMB) was observed in GBM, suggesting other possible causes of aggressiveness (Fig. 1, A5). Correlation analyses revealed that disease fatality was positively correlated with the total number of sCNAs but not TMB (Fig. 1, B1). Generally, copy number deletions were more prominent than amplifications (Fig. 1, B2 & B3). There were more somatic copy number alterations in highly aggressive (Suppl. Fig S1a) than in less aggressive tumors (Suppl. Fig S1b,c, left panel). No such obvious trend was observed for TMB, except for SKCM (Suppl. Fig. S1c, right panel).

### sCNA is associated with cancer onset

Considering, that early cancer onset in some entities is associated with aggressive disease phenotypes, we then asked if sCNAs are equally associated with cancer onset. To address this, we used data from TGCT, in which more than 80% of cases show early onset. We observed more sCNAs in younger compared to older patients (Fig. 1, B4). Given recent reports on gender discrepancies in cancer, we equally investigated possible gender effects in early cancer onset on other cancers with early onset. In younger patients, the female fraction of ACC and GBM were higher. In elderly patients, there was a higher proportion of males with ACC, but a comparable distribution of GBM with females. THYM was predominant in younger males meanwhile GBM and SARC were predominant in young females (Fig. 1, C1). For cancers with early onset, there was no difference in the TMB between males and females (Suppl. Fig. S1d). To further establish the involvement of sCNAs in cancer onset, we specifically looked at adrenocortical carcinoma (ACC). We assessed if females diagnosed with ACC before the age of 45 showed more sCNA alterations than males diagnosed within the same age range. In effect, there were more sCNAs in young females than in males (Fig. 1, C2). In GBM, there was no difference in sCNAs in early and late onset (data not shown). We further investigated additional layers of omics data in early and late GBM patients, as it was the most lethal of all cancers.

### *IDH1* and *TP53* mutations are associated with early onset of GBM

To better understand the molecular basis of GBM aggressiveness and onset, we analyzed somatic mutations as well as sCNAs and gene expression. Early onset of GBM was associated with better overall survival as seen in GBM patients bearing *IDH1* mutations (Fig. 1, C3). High levels of *TP53, IDH1* and *ATRX* mutations were associated with early onset of GBM (Fig. 1, C4). In late onset cases, *PIK3CA* mutations were observed in 7% of cases, but not in early onset cases. Further analysis of sCNAs revealed copy number amplifications of specific oncogenic drivers in late onset, which were absent in early onset (Suppl. Fig. S2a,b). A zoom-in on chromosome 3 revealed sCNA amplification around the *PIK3CA* and *SOX2* gene loci in late onset. It is therefore very likely that early GBM onset is associated with DNA damage, while sCNAs are more prominent in late onset GBM. In support of this, differences in somatic mutation patterns were equally observed for early and late onset in other tumor entities. For example, in sarcoma more than 30% of *TP53* mutations were observed in late onset cases as opposed to less than 10% in early onset (Suppl. Fig. S2c,d). Similarly, in thymoma more than 60% of cases showed mutations in *GTF2I*, while only 18% of early onset cases were affected by this mutation (Suppl. Fig. S2e,f). Lastly, 30% of cases with late onset of TGCT harbored *KIT* mutations, while only 8% of early onset cases were found with *KIT* mutations (Suppl. Fig. S2g,h).

### SOX2 is amplified in aggressive tumors

We identified and annotated all common sCNAs in the 10 most aggressive cancers. (Suppl. Tab. S2 & S3). Differential gene expression analyses comparing the 10 most and the seven least aggressive cancers were then performed (Suppl. Tab. S4). Both data sets were intersected to find gene dosage alterations affecting gene expression (Suppl. Tab. S5). There were more than 50 genes with somatic copy number amplification that were transcriptionally upregulated (Fig. 1, D1) and only five genes with sCNdel that were transcriptionally downregulated. To identify the major players involved in tumor aggressiveness, we classified all affected genes according to their importance in the aggressive disease phenotype using the Boruta package, which is built around the random forest classification algorithm and captures important features that are associated with defined outcome variable in large datasets. As shown in Fig. 1, D2, *SOX2* was the most important variable, followed *by AADAC, FAM83A, UPK1B* and *MYEOV*. Multivariate cox proportional regression performed for the top-20 most important genes revealed that high expression of the genes *SOX2, AADAC, FAM83A, MYEOV, UPKB1, ZIC5, ARL14, LYPD2, CALB1* and *MAGEA12* was associated with poor survival across all analyzed 27 cancer entities (Suppl. Fig. S3). The expression of selected aggressiveness-related genes in poor and better outcome cancers is presented in Fig. 1, D3. We then investigated if *SOX2* as a transcription factor might indeed be regulating the expression of the identified genes. Using published *SOX2* ChIP-seq data (ENDCODE, we found *SOX2* peaks around three of these genes: *AADAC*, *FAM83A* and *MYEOV* (Fig. 1, D4). Individually, Kaplan–Meier analysis of these genes revealed strong association with patient overall survival in all 27 cancer entities (Fig. 1, D5). This suggests that *SOX2* regulates a gene network driving aggressive phenotypes.

### *SOX2 enhances pro-inflammatory signaling *via* FOSL2*

**Fig. 2:**
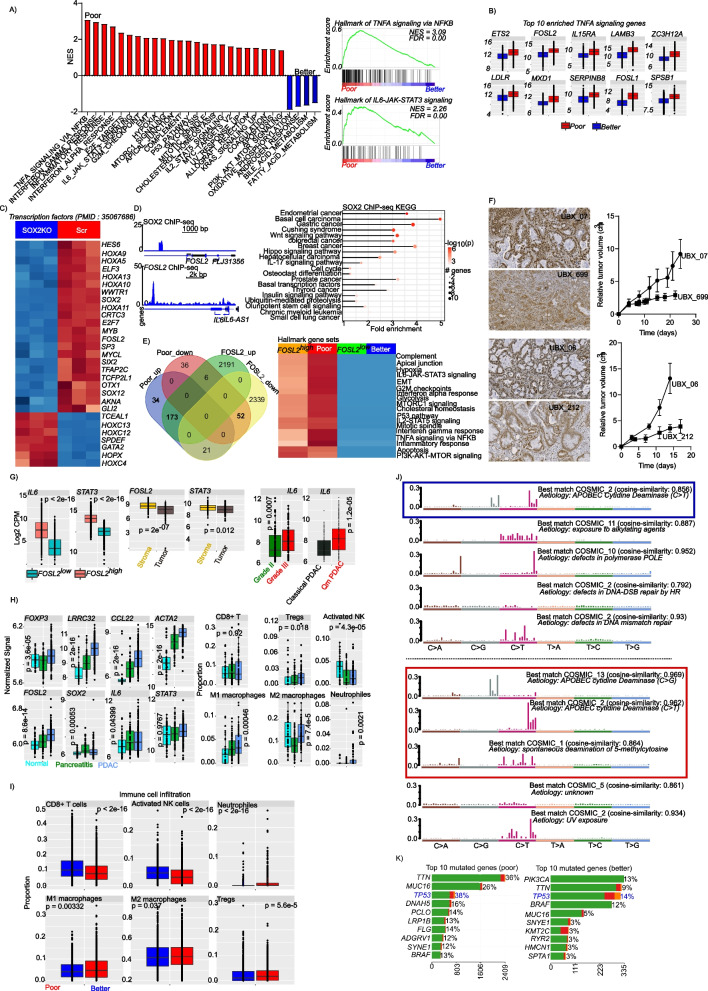
SOX2 promotes disease aggressiveness by enhancing inflammatory and oncogenic signaling via *FOSL2*. (**A**; left panel) A bar plot showing enriched hallmark gene sets in poor and better outcome cancers. Gene sets are considered to be enriched, if the show a false discovery rate of < 0.05. (**A**; right panel) Representative enrichment plot for pro-inflammatory hallmarks gene sets that are enriched in poor outcome cancers. (**B**) Boxplots showing the 10 most enriched gene from the hallmarks of TNFA signaling via NFKB. The hallmarks of TNFA signaling via NFKB was the most significantly enriched hallmark gene set in poor outcome cancers. (**C**) A heatmap showing the expression of significantly differentially expressed transcription factors upon *SOX2* knockdown. (**D**; (left panel) *SOX2* ChIP-sep peak profile showing enrichment around the *FOSL2* gene (upper left panel) and *FOSL2* ChIP-seq peaks around the *IL6* gene (lower left panel) (IL6-JAK-STAT3 signaling is one of the top enriched hallmark gene sets in poor outcome cancers). (**D**; right panel) A pathway plot showing significantly enriched pathways in *SOX2* ChIP-seq data. The ChIP-seq data was derived from the wildtype of the prostate cancer cell lines CWR-R1 and WA01. (**E**; left panel) A Venn diagram showing the intersection between upregulated and downregulated genes in poor out come and better outcome samples and between *FOSL2*^high^ and *FOSL2*^low^ samples. (**E**; right panel) A heatmap comparing enriched hallmark pathways in poor and better outcome cancers and *FOSL2*^high^ and *FOSL2*^low^ samples. *FOSL2*^high^ and *FOSL2*^low^ samples are samples with less than 1000 copies or more than 10,000 copies, respectively. This approach was used investigate if high expression of *FOSL2* is related to the enriched hallmark gene sets seen in the poor outcome cancers. (**F**) FOSL2 staining of PAAD xenografts for fast and slow growing tumors (left panel) and tumor growth curves for the corresponding tumors (right panel). (**G**; left panel) Boxplots showing the expression of the proinflammatory cytokine *IL6* and its downstream effector *STAT3* in *FOSL2* high and low tumors. *FOSL2* high and low groups are the same as described above. (**G**; middle panel) Expression of *FOSL2* and the proinflammatory mediator *STAT3* in different compartments of PAAD tumors. Data is derived from laser microdissected (Maurer et al., 2019) PAAD tumors. The expression of *IL6* was very low in majority of the cases and is not presented. (**G**; right panel) *IL6* gene expression in grade II and grade III PAAD tumors (left panel) and in Qm and classical PAAD subtypes (right panel). Data was generated from resected PAAD samples. (**H**; left panel) Expression of inflammatory mediators and tumor microenvironment marker genes in normal pancreas (*n* = 41), chronic pancreatitis tissue (*n* = 59) and PAAD tissues *n* = 195). (H; right panel) Immune cell proportions derived from deconvolution of gene expression data from normal pancreas tissue, chronic pancreatitis tissue and PAAD tissue. Immune cell proportions were determined with CIBERSORT as implemented in TIMER. (**I**) Boxplots showing immune cell proportions in poor outcome vs better outcome cancers. (**J**) Top five mutational signatures in cancer entities with better outcome (upper panel) and poor outcome (lower panel). (**K**) Top ten most mutated genes in poor outcome cancers (left panel) and better outcome cancers (right panel)

Gene set enrichment analysis revealed strong enrichment in pro-inflammatory pathways in aggressive tumors, such as TNFA signaling via NFkB, *IL6-JAK-STAT3* signaling, inflammatory response and interferon alpha/gamma response, among others (Fig. 2A). Looking at the most enriched hallmark gene set (TNFA signaling via NFkB), the top-10 most enriched genes included two members of the *FOSL* transcription factor family (*FOSL1* and *FOSL2*; Fig. 2B). We focused on these, given that one member of the FOS gene family member (*FOSL1*) was already reported to be associated with GBM aggressiveness [5]. To this end, we investigated if *SOX2* might regulate *FOSL* gene expression. *SOX2* knockdown in the CWR-R1 prostate cancer cell line led to a strong downregulation of *FOSL2*, but not *FOSL1* (Fig. 2C, Suppl. Tab. S6). Analysis of *SOX2* ChIP-seq data revealed that *SOX2* binds to the promoter of *FOSL2*, which in turn regulates *IL6*, a key mediator of cellular inflammation (Fig. 2D, left panel). To confirm the direct involvement of *SOX2* in oncogenic and inflammatory activities, we performed pathway analysis on *SOX2* ChIP-seq data and observed a strong enrichment in pathways driving several cancer entities as well as NFkB-related inflammatory properties (*IL17* signaling pathway; Fig. 2D, right panel). *SOX2* ChIP-seq data was derived from the wildtype of the prostate cancer cell lines CWR-R1 and WA01. The enrichment was calculated as average fold change over input. To establish the involvement of *FOSL2* in disease aggressiveness, we performed differentially gene expression analysis comparing poor and better outcome entities as well as *FOSL*2^high^ and *FOSL2*^low^ tumors using data from all 27 cancer entities. There was a strong overlap between genes upregulated in aggressive tumors and in *FOSL2*^*high*^ tumors (Fig. 2E, left panel; Suppl. Tab. S7). Finally, we investigated if *FOSL2* is driving the enriched gene sets. To this end, we again performed gene set enrichment analysis in *FOSL2*^*high*^ and *FOSL2*^*low*^ (> 10,000 and < 1,000 transcripts, respectively) tumor samples from all 27 cancer entities and compared the enriched hallmark gene sets. As shown in Fig. 2E, right panel, most of the gene sets enriched in the highly aggressive tumors were equally enriched in the *FOSL2*^*high*^ tumors.

### FOSL2 overexpression is associated with aggressive tumors

Upon the observation of high *FOSL2* gene expression in aggressive tumors, we investigated this association in patient specimens and PAAD tumor models. To this end, we stained for FOSL2 protein expression in tumor sections from GBM, PAAD, merkel cell carcinoma (MCC) and PRAD. Additionally, we generated five PAAD xenografts from primary resected patient tumor materials and monitored tumor growth for about 3 weeks. We observed, that fast growing PAAD xenografts showed an overexpression of FOSL2 compared with slow growing tumors, although FOSL2 was expressed in all tumors (Fig. 2F). Furthermore, we compared the expression of FOSL2 in tumor samples from different entities and observed a high expression in tumors from GBM, PAAD and MCC compared with prostate cancer tumors (Suppl. Fig. S4). These findings further support the implication of FOSL2 in tumor aggressiveness. To further investigate the relevance of the *SOX2/FOSL2* axis in disease aggressiveness, we extracted the SOX2 segment mean from all cancer entities and determined a threshold associated with better overall survival. Patient with higher *SOX2* mean segment copy had poor overall survival, irrespective of the entity considered (suppl. Fig. S5a). Using this threshold, we determined that in most aggressive cancers, majority of patients had a *SOX2* segment mean above the threshold (Suppl. Fig. S5b, upper panel). Given that TGCT and PRAD, which are less aggressive entities had relatively higher patient fraction with high *SOX2* segment mean, we estimated the mean *SOX2* segment mean and found, that the mean *SOX2* segment mean for these two entities was very low, indicating a rather stable *SOX2* dosage slightly above our determined threshold (Suppl. Fig. S5b, lower panel). We then evaluated the expression of *SOX2* and *FOSL2* in different subtypes of lung and brain tumors. In lung cancer, higher expression of *SOX2* and *FOSL2* was observed in the most aggressive subtypes of lung cancer basaloid and squamous cell carcinomas (Suppl. Fig. S5c,d). The lung cancer dataset was previously published by *Rousseaux *et al., 2011 (PMID: 23698379, GSE 30219). Similarly, in tumors of the central nervous system, *SOX2* and *FOSL2* expression was higher in pilocytic astrocytoma, ependymoma and glioblastoma multiform compared with medulloblastoma (Suppl. Fig. S5e,f). A subset of medulloblastomas show high expression of *SOX2*, suggesting a subpopulation of patients with aggressive disease. The gene expression used was previously published by Griesinger et al., 2013 (PMID: 24078694, GSE 50161). In bladder cancer, data from patients who developed metastases after tumor resection was analyzed. As shown in Suppl. Fig. S5g, there was no different in patients who developed metastases after resection, compared with those who had localized disease. The data used was previously published by Rose et al., 2013 (PMID: 24145624, GSE51066). A closer look at the top 10 most amplified and top 10 most deleted cases for the *SOX2* locus for each cancer entity revealed, very high *SOX2* dosage in the aggressive cancers (Suppl. Fig. S6).

### Inflammation induces immunosuppression and cytidine deamination

We observed a high expression of *IL6* and its downstream effector *STAT3* in aggressive tumors and in *FOSL2*^*high*^ tumors (Fig. 2G, left panel). We used gene expression data from laser microdissected PAAD samples obtained from the study by Maurer et al., 2019 (PMID 30658994) to determine the tumor compartment driving the observed inflammation. High expression of *FOSL2* and *STAT3* were observed in the stroma (Fig. 2G, middle panel). *IL6* expression was very low in this laser micro-dissected data set and is not presented. Given this difficulty, we used our in-house data from bulk PAAD tissue (E-MTAB-1791, Jandaghi et al., 2016 PMID: 27578530) for further investigations. We focused on PAAD, because of its aggressiveness and presence of dense fibrotic stroma, which could play an important role in inflammation. We therefore analyzed the expression of *IL6* in grade II and grade III PAAD (Fig. 2G, right panel) as well as in quasi-mesenchymal and classical PAAD (Fig. 2G, right panel). As expected, *IL6* was significantly upregulated in grade III as well as in quasi-mesenchymal PAAD tumors (the more aggressive PAAD subtype). We then investigated the expression of immune cell markers in our cohort of almost 300 samples including 41 normal pancreas tissues, 59 chronic pancreatitis tissues and 195 resected PAAD tissues. We observed a steady increase in the expression of *FOXP3, LRRC32*, as well as *FOSL2* and *IL6* from normal through pancreatitis to PAAD (Fig. 2H, left panel). Given that inflammation is associated with immunosuppression, we evaluated the immune cell proportion in the different PAAD samples and observed significant increase in Tregs, pro-inflammatory M1 macrophages and neutrophils, while activated NK cells decreased from normal pancreas tissue through pancreatitis to PAAD (Fig. 2H, right panel). Similar immune infiltration patterns were observed, when we compared poor and better outcome tumors (Fig. 2I). It is known that prolonged inflammation induces cytosine deamination as well as DNA double-strand breaks and leads to genome instability. We therefore analyzed mutational signatures in aggressive and less aggressive tumors and observed an enrichment in cytosine deamination mutational signatures in the aggressive tumors (Fig. 2J) with a characteristic C > G mutation associated with inflammation. Mutational analysis revealed high rates of *TP53* mutations in the aggressive tumors (Fig. 2K).

## Discussion

We investigated the molecular drivers of cancer aggressiveness across 27 cancers using data from the TCGA database and other publicly available and in-house generated data sets. We observed a significant association between sCNAs and cancer aggressiveness. Somatic copy number alterations fueled genetic heterogeneity and supported the amplification of oncogenes and drug resistance genes in cancer [13], thereby influencing outcome. Copy number deletions were more predominant than copy number amplifications and tumor mutational burden was not associated with tumor lethality, except for SKCM. Somatic mutations analyses revealed high levels of *TP53*, *IDH1* and *ATRX* mutations in younger GBM patients, while late onset was characterized by amplification of dedicated oncogenes such as *PIK3CA*. *IDH1* mutation in GBM have been reported to be associated with better prognosis [14]. Given the association of *IDH1* mutations with younger patients, it is very likely that age contributes to the observed survival benefit. This observation therefore supports the consideration of age in translational studies and clinical trials, especially for targeted therapies.

Integrated multi-omic analysis identified that the transcription factor gene *SOX2* was amplified and transcriptionally upregulated in aggressive cancers. *SOX2* has previously been implicated with cancer development [15]. Apart from *SOX2*, other genes involved in cancer aggressiveness, such as *ZIC5*, *FAM83A*, *AADAC* and *MYEOV*, were among the top most significantly associated genes and were associated with patient outcome. The non-coding RNA transcript *MYEOV* has been shown to drive disease aggressiveness in cancer [16]. Furthermore, *FAM83A* has been associated with enhanced tumor cell proliferation and metastasis by regulating Wnt/ß catenin signaling [17]. Interestingly, we observed *SOX2* binding events around some of these genes, suggesting a *SOX2*-regulated gene network.

Pathway analyses revealed activation of several pro-inflammatory pathways in aggressive tumors, such as *TNFA* signaling via NFkB, inflammatory response and *IL6-JAK-STAT3* signaling, among others. Two genes of the *FOSL* transcription factor family, which was recently implicated in inflammation [18], were among the top enriched genes in the TNFA signature. Investigating possible associations between *SOX2* and the *FOSL* genes, we observed *SOX2* regulatory activity on *FOSL2*, and the latter could in turn regulate *IL6* gene expression, indicating that *IL6* is downstream of *FOLS2*, which itself is a target of *SOX2*. Pathway analysis on *SOX2* ChIP-seq data revealed activation of pathways driving the development of several cancers as well as pro-inflammatory pathways. These observations suggest the implication of *SOX2* in sustaining inflammatory processes in aggressive tumors via *FOSL2* and *IL6*. Tumors with high *FOSL2* expression shared similar pathway activation as aggressive tumors, strongly implicating interconnection between *FOSL2* and cancer aggressiveness.

Aggressive tumors equally showed overexpression of pro-inflammatory cytokines and higher levels of immunosuppression. Prolonged inflammation, principally driven by NFkB, can activate cytidine deamination [8] and lead to a characteristic mutational signature and DNA damage. Indeed, we observed a strong enrichment of cytidine deamination mutational signatures in aggressive tumors, a direct indicator of prolonged inflammation in these tumors. Additionally, *TP53* mutations were more predominant in aggressive tumors. It is plausible, that inflammation-induced DNA damage affect tumor suppressor gene function, thereby promoting genomic instability and releasing the cell cycle brake to unleash uncontrolled proliferation.

## Conclusion

Taken together, our data uncover the implications of a *SOX2*-regulated gene expression network controlling cancer aggressiveness via *FOSL2* by activating and sustaining pro-inflammatory TME leading to DNA damage and genomic instability. Targeting these pro-inflammatory processes might minimize DNA damage and improve patient outcome.

## Supplementary Information

**Additional file 1:** **Suppl. Figure S1. **Highly aggressive cancers show higher numbers of CNAs and slightlyhigher tumor mutational burden.(A) CNA plots for top three most aggressive and prevalent cancer. (B) CNA plotsfor top three least aggressive and prevalent cancers. (C) Bar plot showingtumor mutational burden for different age groups for all cancer with more than10% of cases diagnosed before 45 years of age except those shown in previousfigures. (D) A bar plot show the number of CNAs identified in each of theanalyzed cancer entities (left panel) and a bar plot show the tumor mutationalburden in each of the analyzed cancer entities (right panel).

**Additional file 2:** **Suppl. Figure S2. **Somatic gene mutations are prevalent in elderly patients thanyounger patients. (A)A circos plot showing somatic mutations and copy number alteration inglioblastoma patients diagnosed after the age of 45 years (left panel) and azoom on chromosome 3 showing amplification at the *SOX2* gene locus (right panel). (B) A circos plot showing somaticmutations and copy number alteration in glioblastoma patients diagnosed beforethe age of 45 years (left panel) and a zoom on chromosome 3 showing noamplification at the *SOX2* gene locus(right panel) as seen in older patients. (C) An oncoprint showing somaticmutations in sarcoma patients diagnosed before the age of 45 years. (D) Anoncoprint showing somatic mutations in sarcoma patients diagnosed after the ageof 45 years. (E) An oncoprint showing somatic mutations in thymoma patientsdiagnosed before the age of 45 years. (F) An oncoprint showing somaticmutations in thymoma patients diagnosed after the age of 45 years. (G) Anoncoprint showing somatic mutations in patients with testicular germ celltumors diagnosed before the age of 45 years. (H) An oncoprint showing somaticmutations in patients with testicular germ cell tumors diagnosed after the ageof 45 years.

**Additional file 3:** **Suppl. Figure S3. **Genes with somatic copy number amplifications and concomitantupregulation are associated with patient outcome. A forest plot showing multivariatecox proportional hazards regression for genes with somatic amplification andtranscriptional upregulation in poor outcome cancers. 

**Additional file 4:** **Suppl. Figure S4.** FOSL2protein staining in aggressive (GBM, PAAD, MCC) tumors and less aggressivetumors PRAD)

**Additional file 5:** **Suppl. Fig. S5. ***SOX2* gene dosage isassociated with overall survival across multiple cancer entities. A) A Kaplan-Meier overall survivalcurve for patients with high and low *SOX2*mean copy segment. Samples were dichotomized using the survminer R package.Mean segment low and mean segment high are *SOX2*CNA segments as determined by the gaia Bioconductor package. B) A bar plotshowing the percentage of patient in each cancer entity with mean *SOX2* segment mean higher or lower thanthe cut-off determined in A. C) Bar plots showing the expression of *SOX2* in different molecular subtypes oflung cancer as well as non-tumor lung tissue (normal). Large cellneuroendocrine (LCNE), basaloid (BAS), adenocarcinoma (ADK), squamous cellcarcinoma (SQC), carcinoid (CARCI) and small cell carcinoma (SCC). D) Bar plots showing the expression of *FOSL2* in different molecular subtypes oflung cancer as well as non-tumor lung tissue. E) Bar plots showing theexpression of *SOX2* in differentmolecular subtypes of CNS tumors as well as non-tumor brain tissue. F) Barplots showing the expression of *FOSL2*in different molecular subtypes of CNS tumors as well as non-tumor braintissue. G) Bar plots showing the expression of *FOSL2* in prostate cancer patients who developed either localized ormetastatic disease after resection.

**Additional file 6:** **Suppl. Fig. S6.** A waterfallplot showing the mean *SOX2* genedosage in the top 10 cases with the most amplified and the most deleted *SOX2* alterations in all cancer entities.

**Additional file 7.**

## Data Availability

All data used in this manuscript is publicly available through TCGA or under the given accession number of GEO. Data supporting our findings are reported in the manuscript and the supplementary information.

## Abbreviations

ACC: Adrenocortical carcinoma
BLCA: Bladder Urothelial Carcinoma
LGG: Brain Lower Grade Glioma
BRCA: Breast invasive carcinoma
CESC: Cervical squamous cell carcinoma and endocervical adenocarcinoma
CHOL: Cholangiocarcinoma
COAD: Colon adenocarcinoma
ESCA: Esophageal carcinoma
GBM: Glioblastoma multiforme
HNSC: Head and Neck squamous cell carcinoma
KICH: Kidney Chromophobe
KIRC: Kidney renal clear cell carcinoma
KIRP: Kidney renal papillary cell carcinoma
LIHC: Liver hepatocellular carcinoma
LUAD: Lung adenocarcinoma
LUSC: Lung squamous cell carcinoma
OV: Ovarian serous cystadenocarcinoma
PAAD: Pancreatic adenocarcinoma
PRAD: Prostate adenocarcinoma
SARC: Sarcoma
SKCM: Skin Cutaneous Melanoma
STAD: Stomach adenocarcinoma
TGCT: Testicular Germ Cell Tumors
THYM: Thymoma
THCA: Thyroid carcinoma
UCEC: Uterine Corpus Endometrial Carcinoma
UVM: Uveal Melanoma
EMT: Epithelial to mesenchymal transition
TNFA: Tumor necrosis factor alpha
IL: Interleukin
TMB: Tumor mutational burden
sCNA: Somatic copy number alteration
sCNAmp: Somatic copy number amplification
sCNdel: Somatic copy number deletion
TCGA: The cancer genome atlas
GEO: Gene expression omnibus
FDR: False discovery rate
